# Radiomics Signature Based on Support Vector Machines for the Prediction of Pathological Complete Response to Neoadjuvant Chemoradiotherapy in Locally Advanced Rectal Cancer

**DOI:** 10.3390/cancers15215134

**Published:** 2023-10-25

**Authors:** Chao Li, Haiyan Chen, Bicheng Zhang, Yimin Fang, Wenzheng Sun, Dang Wu, Zhuo Su, Li Shen, Qichun Wei

**Affiliations:** 1 Department of Radiation Oncology (Key Laboratory of Cancer Prevention and Intervention, China National Ministry of Education, Key Laboratory of Molecular Biology in Medical Sciences), The Second Affiliated Hospital, Zhejiang University School of Medicine, Hangzhou 310009, China; chao_li@zju.edu.cn (C.L.); chenhaiyan@zju.edu.cn (H.C.); bicheng_zhang@zju.edu.cn (B.Z.); sunwenzheng@zju.edu.cn (W.S.); wudang@zju.edu.cn (D.W.); 2517018@zju.edu.cn (Z.S.); 2 Department of Colorectal Surgery and Oncology (Key Laboratory of Cancer Prevention and Intervention, China National Ministry of Education, Key Laboratory of Molecular Biology in Medical Sciences), The Second Affiliated Hospital, Zhejiang University School of Medicine, Hangzhou 310009, China; 21918527@zju.edu.cn

**Keywords:** radiomics signature, nomogram, pathological complete response (pCR), neoadjuvant chemoradiotherapy (nCRT), locally advanced rectal cancer (LARC)

## Abstract

**Simple Summary:**

This study developed CT-based radiomics signatures using the least absolute shrinkage and selection operator (LASSO), random forest (RF) and support vector machine (SVM) algorithms to predict the pathological complete response (pCR) in locally advanced rectal cancer (LARC) patients who underwent neoadjuvant chemoradiotherapy. Among these methods, the SVM-based radiomics score (Radscore) exhibited superior performance compared to the others, with area under the receiver operating characteristic curves (AUCs) of 0.880 and 0.830 in the training and validation cohorts, respectively. By integrating the SVM-based Radscore with clinical indicators, a nomogram was created for predicting pCR, achieving AUCs of 0.910 and 0.866 in the training and validation cohorts, respectively. The study highlighted the promising performance of the SVM-based Radscore and the value of the radiomics nomogram for predicting pCR in LARC patients. Additionally, the identification of an optimal radiomics signature can significantly improve the accuracy of pCR prediction.

**Abstract:**

The objective of this study was to evaluate the discriminative capabilities of radiomics signatures derived from three distinct machine learning algorithms and to identify a robust radiomics signature capable of predicting pathological complete response (pCR) after neoadjuvant chemoradiotherapy in patients diagnosed with locally advanced rectal cancer (LARC). In a retrospective study, 211 LARC patients were consecutively enrolled and divided into a training cohort (*n* = 148) and a validation cohort (*n* = 63). From pretreatment contrast-enhanced planning CT images, a total of 851 radiomics features were extracted. Feature selection and radiomics score (Radscore) construction were performed using three different machine learning methods: least absolute shrinkage and selection operator (LASSO), random forest (RF) and support vector machine (SVM). The SVM-derived Radscore demonstrated a strong correlation with the pCR status, yielding area under the receiver operating characteristic curves (AUCs) of 0.880 and 0.830 in the training and validation cohorts, respectively, outperforming the RF and LASSO methods. Based on this, a nomogram was developed by combining the SVM-based Radscore with clinical indicators to predict pCR after neoadjuvant chemoradiotherapy. The nomogram exhibited superior predictive power, achieving AUCs of 0.910 and 0.866 in the training and validation cohorts, respectively. Calibration curves and decision curve analyses confirmed its appropriateness. The SVM-based Radscore demonstrated promising performance in predicting pCR for LARC patients. The machine learning-driven nomogram, which integrates the Radscore and clinical indicators, represents a valuable tool for predicting pCR in LARC patients.

## 1. Introduction

Locally advanced rectal cancer (LARC) is a prevalent malignancy that is encountered globally. The standard treatment for LARC involves total mesorectal excision (TME) surgery following neoadjuvant chemoradiotherapy (nCRT), resulting in pathologic complete response (pCR) in approximately 15% to 27% of patients [1,2,3]. Controversially, the “watch and wait” strategy has emerged as a preferred option due to reduced surgical risks and improved postoperative quality of life in patients achieving a complete response [4,5]. Currently, pCR can only be determined through the histopathologic examination of surgically resected specimens. Therefore, the critical challenge lies in developing a non-invasive and accurate method to identify complete response after chemoradiotherapy.

Medical imaging techniques such as positron emission tomography (PET), endorectal ultrasound (EUS) and magnetic resonance imaging (MRI) have been utilized as non-invasive methods to assess the response to CRT and evaluate pCR [6,7,8,9,10,11]. PET/CT can effectively detect abnormal metabolic activity and metastases, and the features extracted from PET/CT imaging have shown potential in predicting treatment response. EUS allows the direct visualization of the rectal wall and adjacent structures, making it valuable in predicting response to CRT. Similarly, MRI holds promise in predicting responses to CRT due to its significant role in cancer diagnosis and staging. Different imaging modalities have been employed to assist in predicting pCR, yielding promising results. However, accessing these additional imaging modalities such as PET, EUS and MRI often requires more time and effort compared to CT imaging. Moreover, CT imaging plays a crucial role in radiotherapy planning for LARC patients. It provides detailed anatomical information, allowing for the accurate delineation of the gross tumor volume (GTV) in the rectal region. This delineation is essential for precise targeting during radiotherapy delivery. Additionally, CT imaging facilitates the extraction of radiomics features, which are quantitative metrics derived from medical images. These features capture tumor heterogeneity and pathophysiological characteristics and can be used for predictive modeling and treatment planning.

Recent studies in radiomics have highlighted the remarkable potential of CT-based radiomics analyses in predicting the response to CRT in LARC, as evidenced by numerous references [12,13,14,15,16,17,18,19,20]. The majority of these studies primarily focused on predicting the pCR status [12,13,14,15,16,17,18]. Despite these valuable contributions, the current body of research still faces several limitations that need to be addressed. One of the prominent challenges is related to the quality and characteristics of the datasets utilized. This includes the lack of independent validation datasets [13,15], inadequately small sample sizes [12,15] and the presence of imbalanced test datasets [17]. Despite achieving exceptional performance on the training datasets, these models often exhibit significant underperformance when tested on independent validation datasets [16]. Consequently, concerns arise regarding the robustness and generalizability of the developed models. Another critical gap in the existing literature is the insufficient integration of clinical indicators to enhance response prediction [12,13,14,15]. Incorporating relevant clinical factors in combination with radiomic features can provide a more comprehensive understanding of the predictive models, yielding more accurate and reliable results. Furthermore, it is paramount to explore a robust classifier to ensure the development of reliable predictive models [18]. Different machine learning algorithms should be evaluated to identify the most suitable and effective approach for predicting pCR in LARC based on radiomics scores.

The radiomics score (Radscore), which is calculated by assigning weights to selected features based on their respective coefficients, has been employed in prior research to predict the response to CRT in patients with LARC [16,18,21,22]. However, to the best of our knowledge, no study has comprehensively evaluated its feasibility and effectiveness in combination with diverse machine learning techniques. This research gap emphasizes the need for further investigations to leverage the potential synergies between Radscores and machine learning techniques (LASSO, RF and SVM), aiming to advance the accuracy and applicability of predictive models for pCR prediction in LARC. Therefore, the purpose of our study was to develop and validate a robust radiomics nomogram that incorporates the Radscore derived from planning CT scans, along with clinical indicators, for the preoperative prediction of pCR in patients with LARC. Our work aims to non-invasively evaluate patient outcomes and assist LARC patients in potentially avoiding surgery through the use of a radiomics signature obtained from planning CT scans.

## 2. Materials and Methods

### 2.1. Patients

This retrospective study was approved by our institutional review board. We consecutively enrolled patients diagnosed with LARC who underwent CRT and subsequent surgery at the Second Affiliated Hospital, Zhejiang University School of Medicine, from October 2015 to June 2021. The patient recruitment process, along with the inclusion and exclusion criteria, are summarized in Figure S1.

The inclusion criteria for this study were as follows: (1) LARC patients diagnosed with histologically confirmed rectal adenocarcinoma; (2) patients who underwent long-course neoadjuvant CRT followed by TME surgical resection; (3) patients with available pretreatment contrast-enhanced planning CT images acquired using the same CT scanner. On the other hand, the exclusion criteria were: (1) patients whose surgery was either canceled or delayed for more than 8 months after completing CRT; (2) patients diagnosed with non-adenocarcinoma LARC; (3) patients with inadequate clinicopathological data or CT images of poor quality.

A total of 211 consecutive patients were enrolled in this study, and they were divided into two datasets in a ratio of 7:3 using computer-generated random numbers. The training cohort consisted of 148 patients, while the validation cohort included 63 patients. The allocation was conducted in a manner that maintained a balanced distribution of pCR rates between the two cohorts. Baseline clinical characteristics, such as age, gender, body mass index (BMI), T stage, N stage, distance from anal verge, tumor volume, tumor diameter, tumor length, prescription dose, levels of carcinoembryonic antigen (CEA) and carbohydrate antigen 19-9 (CA19-9), were extracted from the patients’ pretreatment medical records (Table S1).

### 2.2. Standard of Reference

All patients included in this study underwent intensity-modulated radiation therapy (IMRT) with standard contouring. The total radiation dose administered was either 50 Gy (delivered in daily fractions of 2.0 Gy) or 57.5 Gy (delivered in daily fractions of 2.3 Gy), administered 5 days a week over a duration of 5 weeks. Concurrently, chemotherapy was administered alongside radiation therapy using capecitabine at a dose of 825 mg/m^2^ orally twice daily on radiation therapy days. Then, patients receive capecitabine alone or CAPEOX chemotherapy for two cycles before surgery. TME surgery was performed within 12 weeks of the completion of neoadjuvant CRT.

**Figure 1:**
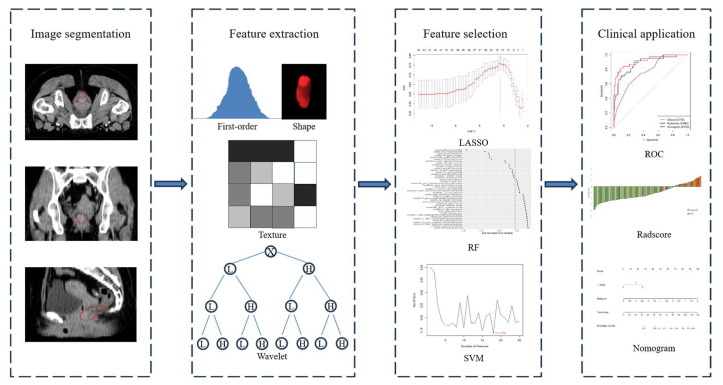
Radiomics workflow adopted in our study, encompassing the following steps: (I) Manual segmentation of tumors was performed on the planning CT images, delineating the gross tumor volume (GTV) with a red circle. (II) A total of 850 radiomics features were extracted from the defined region of interest. (III) Radiomics feature selection techniques were employed to identify the most informative features. (IV) A radiomics nomogram was developed by integrating the radiomics score, calculated from the selected radiomics features, and relevant clinical indicators.

The surgical resection specimens were meticulously evaluated by specialized gastrointestinal pathologists who were blinded to the patients’ clinical and CT findings. Pathological tumor staging was conducted in accordance with the American Joint Committee on Cancer (AJCC) TNM system. The response to CRT was assessed using the tumor response grading (TRG) system. Based on their response, patients were then categorized into two groups: pCR and non-pCR. pCR was defined as the absence of viable tumor cells in both the primary tumor and the associated lymph nodes. The study workflow is depicted in Figure 1.

### 2.3. Feature Extraction

All patients in the study underwent a simulation CT scan utilizing the LightSpeed RT system (GE Healthcare, Chicago, IL, USA), a 16-channel multi-detector row CT scanner specifically designed for radiotherapy treatment planning. These scans involved the administration of contrast-enhanced CT images following the intravenous injection of 80–100 mL of iodinated contrast material (Medrad, Bayer, Leverkusen, Germany) at a controlled rate of 2.0–3.0 mL/s. For each patient, images were acquired during the portal venous phase with a fixed delay time of 50 s. The acquired CT images were subsequently reconstructed to provide a pixel matrix of 512 × 512 and a slice thickness of 2.5 mm, ensuring precise visualization and analysis. To mitigate any potential variability arising from parameters related to voxel size, radiomics data were extracted from images that had been resampled to isometric voxels measuring 1 × 1 × 1 mm^3^. This standardized voxel size allowed consistent and comparable radiomics analysis across the dataset.

The primary tumors were independently contoured manually by two experienced abdominal radiation oncologists at our institution. Staging MR T2-weighted sequences were utilized as a supplementary tool to assist in target definition. To assess the reliability and consistency of the extracted features, intra- and interclass correlation coefficients (ICCs) were employed. Features with ICC values exceeding 0.75 were deemed suitable for further analysis in this study. Subsequently, the CT images and GTV structures were retrieved from the Eclipse Treatment Planning System (TPS), developed by Varian Medical System, and then transferred to an external workstation running auto-segmentation software named AccuContour 3.2 (Manteia, Milwaukee, WI, USA). This software allowed the automatic calculation of radiomics features based on the provided CT images and GTV structures.

A total of 851 imaging features were extracted from the planning CTs for each patient. These features were classified into several categories, which included 18 first-order features, 14 shape features, 24 grey-level co-occurrence matrix (GLCM) features, 16 grey-level run-length matrix (GLRLM) features, 16 gray-level size zone matrix (GLSZM) features, 14 gray-level dependence matrix (GLDM) features, and 5 neighbor gray-level difference matrix (NGTDM) features. Additionally, these categories comprised 744 high-dimensional feature types, encompassing both the first-order features and texture features calculated from the images using a wavelet filter with a three-step depth of decomposition. This comprehensive analysis involved considering various combinations of wavelet decompositions, specifically HHH, HHL, HLH, HLL, LHH, LHL, LLH and LLL (Table S4). Following feature extraction, all radiomics features were normalized using a z-score transformation method. This involved transforming the data into standardized intensity ranges for each imaging modality across all subjects, resulting in a mean of 0 and a standard deviation of 1.

### 2.4. Feature Selection and Radscore Construction

To construct a robust radiomics signature and select the optimal radiomics features from the initial pool of 851 radiomics features, we employed three feature selection steps. Initially, we conducted Pearson correlation analysis with an r threshold of 0.8 to identify and eliminate redundant features. Subsequently, features were selected based on univariate logistic regression between the pCR and non-pCR groups in the training cohort, using a threshold of 0.1 to avoid confounding effects during multivariate analysis.

In the next step, three commonly used machine learning methods were employed to select key features from the previously identified set: the least absolute shrinkage and selection operator (LASSO), random forest (RF) and support vector machine (SVM). These algorithms were utilized to identify the most relevant features for the task at hand. Radscores, which represent the radiomics scores, were calculated for each patient by linearly combining the selected features and weighting them by their respective coefficients. These Radscores were then used to predict the probability of pCR.

To obtain the LASSO radiomics score, we summed the selected radiomics features weighted by their non-zero coefficients. Features with minimal impact on the target variable were assigned a weight of zero and excluded from further analysis. In order to address potential overfitting issues, we employed the Fast Unified Random Forests for Survival, Regression, and Classification (RF-SRC) feature selection approach. This method ranks the variables based on minimal depth and determines the optimal number of features with the lowest out-of-bag (OOB) error. The RF radiomics score was obtained by summing the selected radiomics features and weighting them according to their importance derived from the RF-SRC method [23,24,25,26]. Additionally, the SVM-based Recursive Feature Elimination (SVM-RFE) algorithm was utilized to determine the optimal subset of features. The SVM-RFE algorithm iteratively removes features with the lowest weights and selects the most relevant features for the classification task. The SVM radiomics score was then calculated by summing the selected radiomics features weighted by their corresponding non-zero coefficients obtained from the linear SVM model [27,28,29,30].

By employing these advanced feature selection techniques and leveraging the importance of variables and coefficients, we were able to generate informative radiomics scores that effectively captured the discriminatory power of the selected radiomic features. These scores contribute to the development of robust classifiers and predictive models in radiomics research, enabling improved accuracy and reliability in various applications.

In the third step, we identified the radiomics classifier with the highest area under the curve (AUC) and accuracy as the optimal Radscore. All three classifiers were trained using 10-fold cross-validation on the training cohort to determine the optimal parameter configuration for each classifier. Subsequently, they were tested on the validation cohort. The performance of each classifier was evaluated using receiver operating characteristic (ROC) curves, and the AUC was used as the metric to assess their performance. Accuracy, sensitivity, specificity, negative-predictive value (NPV), and positive-predictive value (PPV) in both the training and validation sets were calculated based on the Youden index.

### 2.5. Nomogram Integrating Radscore and Clinical Indicators

The Radscore and all the aforementioned clinical candidate predictors were subjected to univariable and multivariable logistic regression analyses in the training cohort. In order to enhance the interpretability and facilitate statistical analysis, we employed a transformation method for continuous variables. Specifically, the continuous clinical indicators were transformed into categorical variables using ROC values. This allowed us to determine the optimal cut-off point for each continuous clinical feature. This categorization of variables aided in simplifying the statistical analysis process while ensuring its robustness and academic integrity.

Subsequently, a radiomics nomogram was developed based on the results of the multivariate logistic analysis. This nomogram was designed to estimate the individual probability of achieving a pCR within the training cohort. To assess the discriminatory ability of the radiomics nomogram, several performance metrics were calculated, including AUC, accuracy, sensitivity, specificity, PPV, and NPV. These metrics provided an evaluation of the nomogram’s effectiveness in distinguishing between different response outcomes and predicting the likelihood of achieving a pCR. To assess the calibration of the radiomics nomogram, a calibration curve was generated by plotting the actual probability of pCR against the predicted probability of pCR from the nomogram. Furthermore, the goodness-of-fit of the radiomics nomogram was evaluated using the Hosmer–Lemeshow test.

In order to evaluate the clinical utility of the radiomics nomogram, a decision curve analysis (DCA) was conducted. Net benefits were calculated at various threshold probabilities to determine the potential value of the nomogram in clinical decision making.

To validate the predictive performance of the radiomics nomogram, the multivariable logistic regression model derived from the training cohort was applied to all patients in the validation cohort. This allowed us to assess the generalizability and robustness of the nomogram in an independent dataset.

### 2.6. Statistical Analysis

All statistical analyses were performed using RStudio version 2022.12.0+353. The following packages in R software were utilized: “caret”, “glmnet”, “rms”, “pROC”, “rmda”, “MASS”, “fmsb”, “ggplot2”, “randomForestSRC”, “ggRandomForests”, “ResourceSelection”, “msvmRFE” and “e1071”. To compare the AUC values of the radiomics signature, a deLong test was employed. Chi-square tests were used to compare the differences in categorical variables (gender, age, BMI, tumor volume, tumor diameter, tumor length, T stage, N stage, CEA and CA19-9 levels, distance from anal verge, prescription dose), and two-sample *t*-test was used to compare the differences in continuous variable (Radscore). All statistical tests were two-sided, and *p*-values below 0.05 were considered statistically significant.

## 3. Results

### 3.1. Clinical Characteristics

In this study, a total of 211 consecutive patients were included, and 50 of them achieved pCR, representing a rate of 23.7%. The distribution of the pCR rate was similar in the training and validation cohorts (*p* = 0.390). Additionally, other pretreatment clinical characteristics, such as gender, age, BMI, tumor volume, tumor diameter, tumor length, T stage, CEA and CA19-9 levels, distance from anal verge, and prescription dose, were balanced between the cohorts, with the exception of N stage (*p* = 0.025). Furthermore, there were no significant differences in these clinical characteristics between the pCR and non-pCR groups, except for tumor volume within the training cohort (*p* = 0.005). For a detailed summary of the clinical characteristics, please refer to Table 1.

### 3.2. Feature Selection and Radscore Construction

A total of 851 imaging features were extracted from the pretreatment planning CT images. To construct the Radscore using the aforementioned dataset, we initially performed Pearson correlation analysis and univariate logistic regression in the training cohort. This process resulted in the selection of 44 features as potential predictors. Subsequently, the LASSO, RF and SVM algorithms were trained using these selected features, and their differentiation abilities were evaluated in the validation cohort.

**Figure 2:**
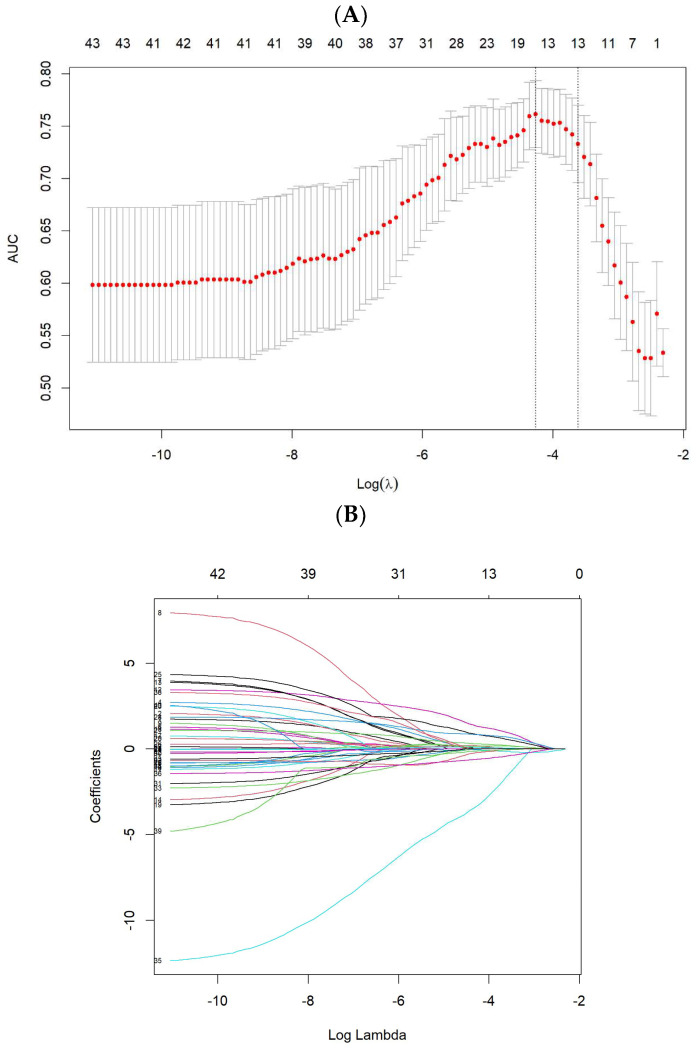

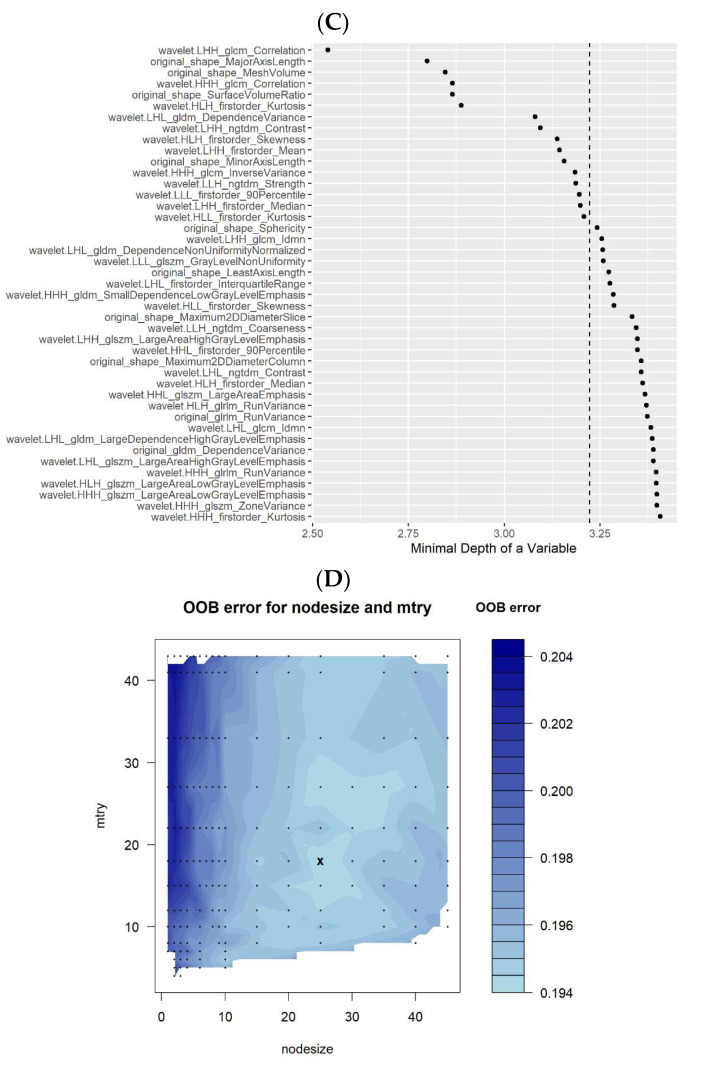

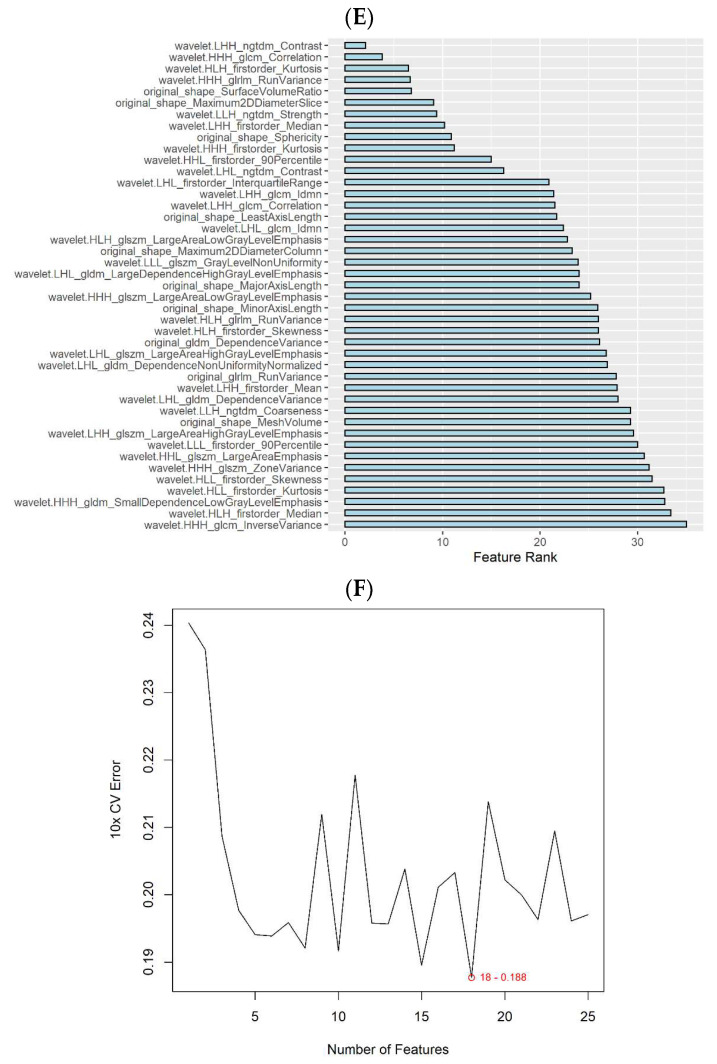
Development process of the three algorithms utilized in our study. (**A**) LASSO feature selection was performed using a minimum criteria approach. The optimal tuning parameter λ was determined to be 0.014, and 14 non-zero coefficients were selected. (**B**) The LASSO coefficient profiles of the radiomics features were examined. (**C**) RF feature selection was conducted based on the minimal depth approach, resulting in the selection of 16 features with depth higher than 3.222. (**D**) The optimal tuning parameters (x point) for the RF were determined as mtry = 18 and nodesize = 25 using the out-of-bag error. (**E**) SVM feature selection was employed to identify the most informative features using the feature rank approach. The optimal tuning parameters, gamma and cost, were determined to be 1 and 10, respectively. (**F**) A total of 18 features were selected based on evaluation of the cross-validation error. LASSO: least absolute shrinkage and selection operator; RF: random forest; SVM: support vector machines.

The LASSO was used to identify the optimized subset of features and calculate the Radscore for each patient. Fourteen features with non-zero coefficients were identified using minimum criteria (Figure 2A,B). For the RF algorithm, 16 features were chosen based on the minimal depth criterion in the training cohort (Figure 2C,D). The SVM algorithm selected the top 18 features with the highest accuracy for pCR prediction (Figure 2E,F). The coefficients and the calculation formula for the Radscores are provided in Table S3. Consequently, the Radscores were calculated for each patient in both the training and validation cohorts (Figure S2A–C).

**Figure 3:**
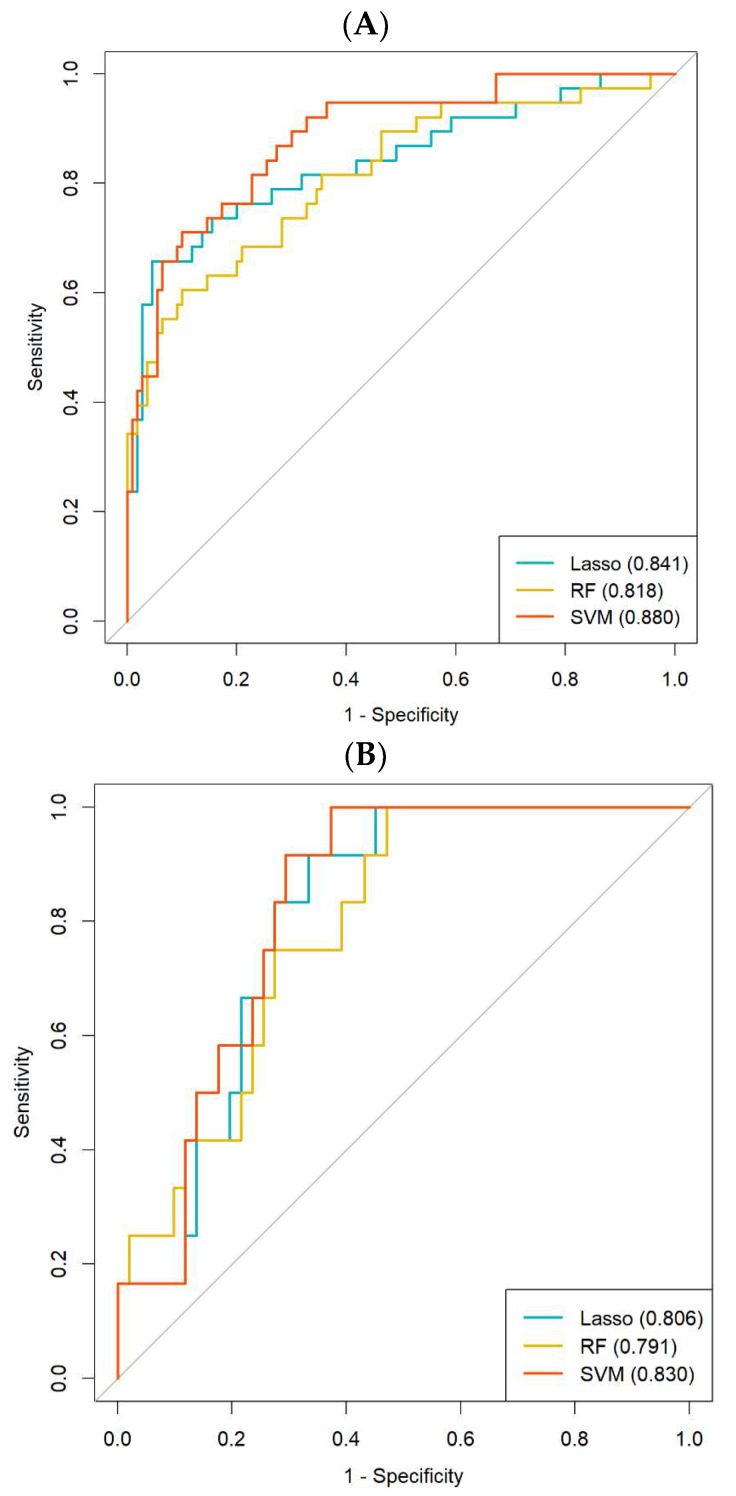

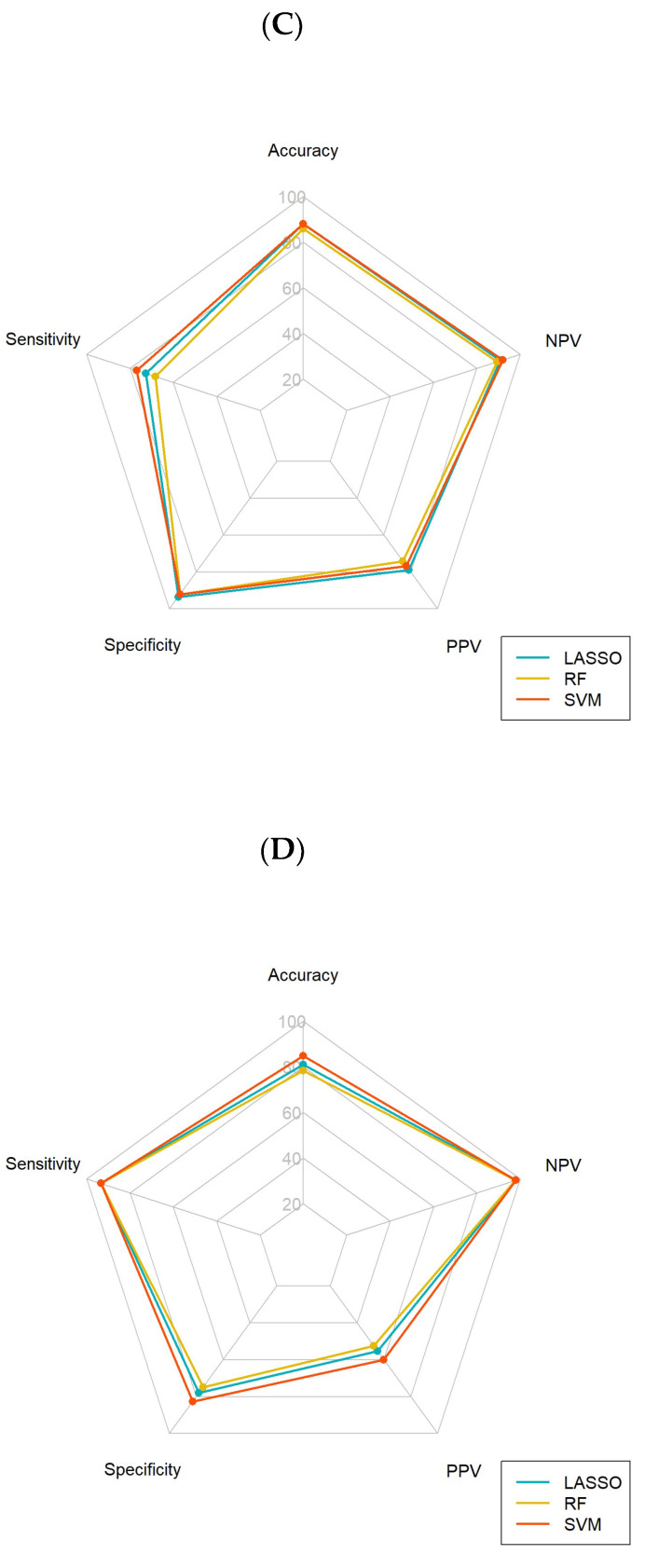
Predictive performance evaluation of the three methods, namely LASSO, RF and SVM, in both the training and validation cohorts. (**A**) ROC curves were plotted to visualize the performance in the training cohort. (**B**) Similarly, ROC curves were generated to assess the performance in the validation cohort. (**C**) The accuracy, sensitivity, specificity, NPV and PPV of the LASSO, RF and SVM were evaluated in the training cohort. (**D**) Likewise, the accuracy, sensitivity, specificity, NPV and PPV of the three classifiers were assessed in the validation cohort. LASSO: least absolute shrinkage and selection operator; RF: random forest; SVM: support vector machines; ROC: receiver operating characteristic; NPV: negative-predictive value; PPV: positive-predictive value.

The Radscore derived from the SVM method demonstrated AUC values of 0.880 (95% confidence interval [CI], 0.823–0.946) and 0.830 (95% CI, 0.722–0.928) in the training and validation cohorts, respectively. Similarly, the Radscore derived from the LASSO and RF methods exhibited AUCs of 0.841 (95% CI, 0.758–0.924) and 0.818 (95% CI, 0.734–0.902) in the training cohort, which were further validated in the validation cohort with AUCs of 0.806 (95% CI, 0.696–0.916) and 0.791 (95% CI, 0.670–0.912). These findings suggest that the Radscore derived from the SVM model demonstrated better predictive efficacy compared to the other two methods (Figure 3A,B).

The accuracy, sensitivity, specificity, NPV and PPV were high for the SVM in both the training and validation cohorts, while specificity and PPV were relatively lower in the training cohort. The radar chart depicted the performance metrics of accuracy, sensitivity, specificity, NPV, and PPV for the three classifiers in both the training and validation cohorts (Figure 3C,D). For more detailed information on the performance of the Radscore, please refer to Table S2.

**Figure 4:**
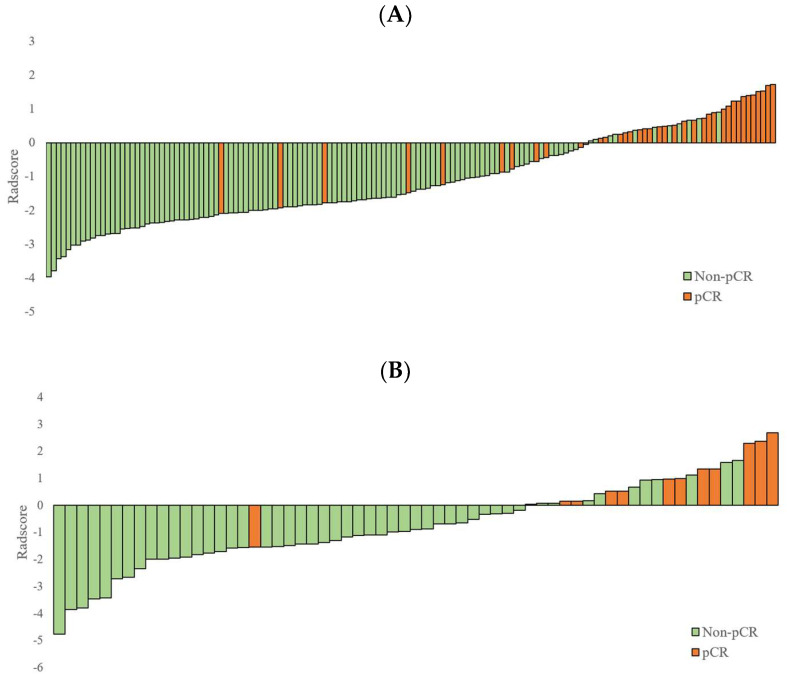
Performance evaluation of the radiomics score (Radscore) calculated by the SVM, assessing its predictive abilities in both the training and validation cohorts. (**A**) The Radscore for each patient in the training cohort is illustrated, demonstrating the distribution and variation in the Radscore values. (**B**) Similarly, the Radscore for each patient is visualized in the validation cohort. SVM: support vector machines.

The Radscore derived from the SVM was significantly higher in the pCR group compared to the non-pCR group in both the training cohort (−0.2601 ± 1.1362 vs. −1.6742 ± 0.8722, *p* < 0.0001) and the validation cohort (−0.3133 ± 1.0479 vs. −1.6379 ± 0.9098, *p* < 0.0001) (Table 1, Figure 4).

### 3.3. Nomogram Integrating Radscore and Clinical Indicators

**Figure 5:**
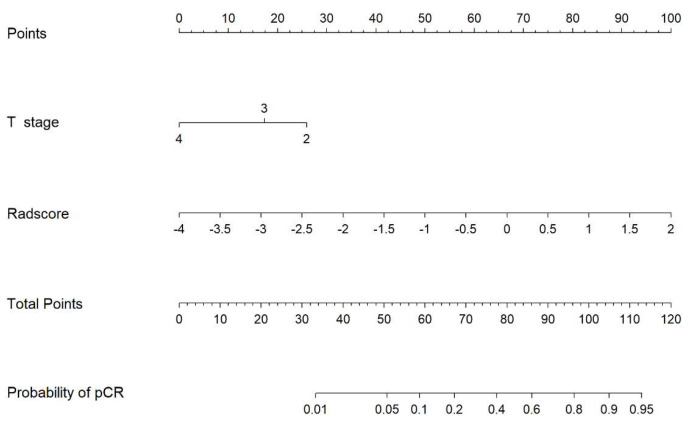
Nomogram that integrates the radiomics score with the clinical indicators in our study.

The Radscore based on a SVM algorithm, along with clinical indicators such as pretreatment T stage, were identified as independent predictors for predicting the pCR status. These results were obtained through univariable and multivariate logistic regression analyses, as shown in Table 2. Subsequently, we constructed a radiomics nomogram incorporating the independent predictor, as illustrated in Figure 5.

**Figure 6:**
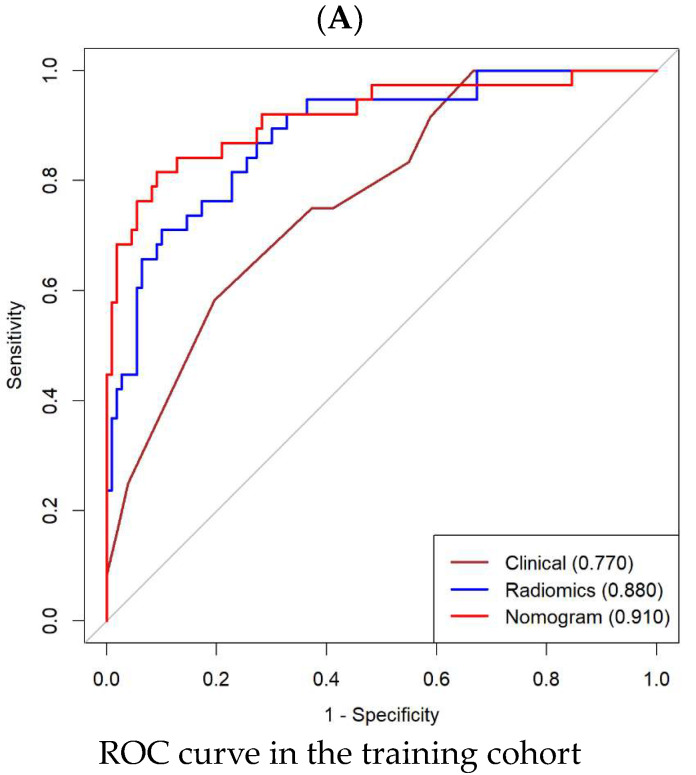

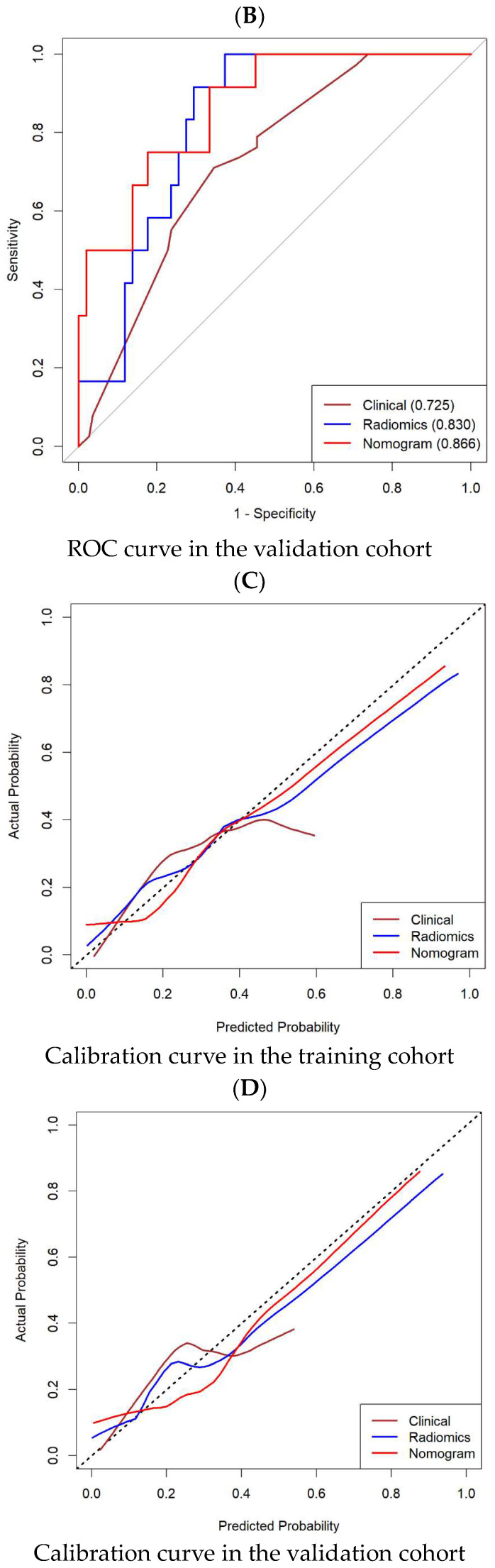

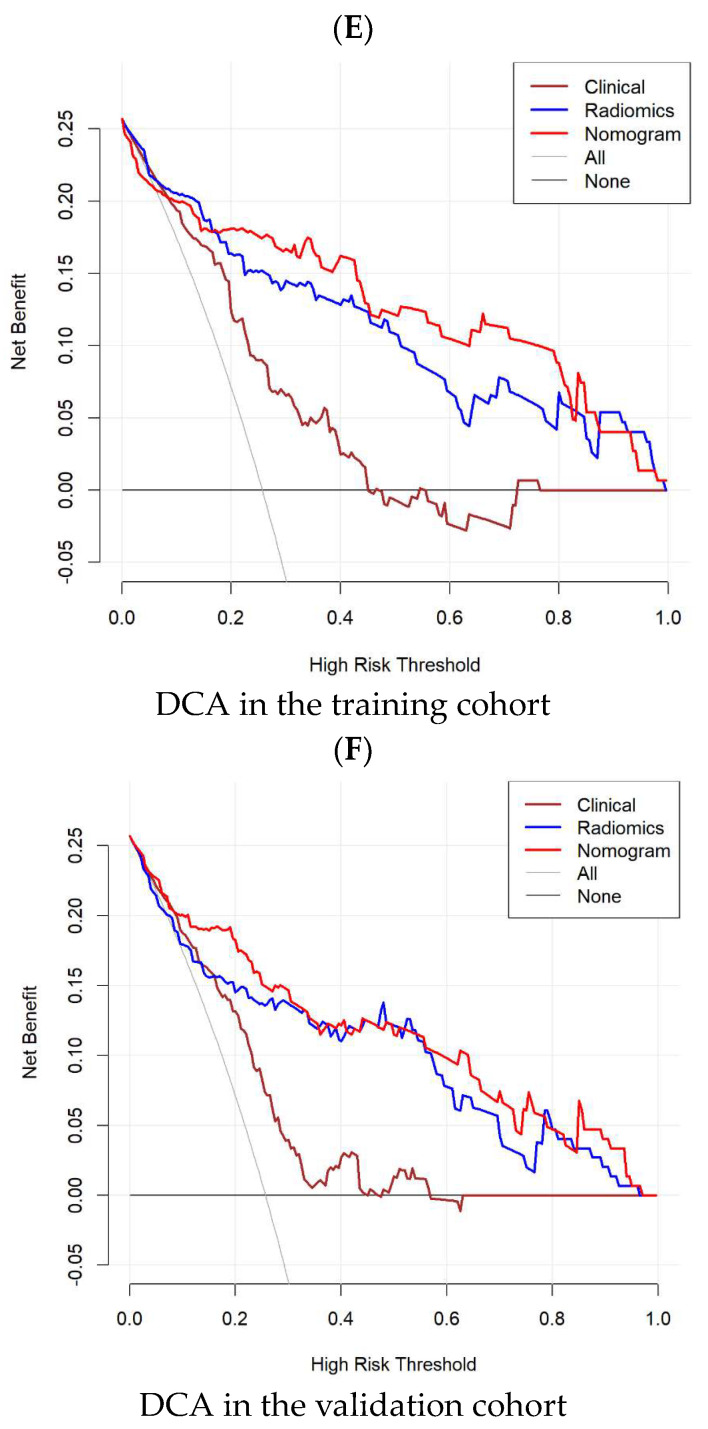
Performance evaluation of the clinical, radiomics, and nomogram models in both the training and validation cohorts. (**A**) ROC curves were plotted to assess and compare the discriminatory power of the clinical, radiomics and nomogram models in the training cohort. (**B**) Similarly, ROC curves were generated to evaluate the discriminatory abilities of these models in the validation cohort. (**C**) Calibration curves were constructed to examine the agreement between the predicted and observed outcomes of the clinical, radiomics and nomogram models in the training cohort. (**D**) Likewise, calibration curves were analyzed to assess the agreement between predicted and observed outcomes in the validation cohort for all three models. (**E**) DCA was performed to evaluate the clinical utility and net benefit of the clinical, radiomics and nomogram models in the training cohort. (**F**) Additionally, DCA was conducted to assess the clinical utility and net benefit of these models in the validation cohort. ROC: receiver operator characteristic; DCA: decision curve analysis.

The AUC for the probability of achieving pCR derived from the radiomics nomogram was 0.910 (95% CI, 0.815–0.976) in the training cohort and 0.866 (95% CI, 0.762–0.970) in the validation cohort. For comparison, AUC values of 0.770 (95% CI, 0.629–0.911) in the training cohort and 0.725 (95% CI, 0.642–0.808) in the validation cohort were obtained from the clinical indicators alone. These findings indicate that the radiomics nomogram exhibited superior predictive efficacy compared to both the Radscore alone and the clinical indicators alone, as shown in Figure 6A,B. Furthermore, the Hosmer–Lemeshow test yielded non-significant *p*-values of 0.281 and 0.585 in the training and validation cohorts, respectively, suggesting a good fit of the nomogram to the observed data. Detailed information regarding the performance of the three models can be found in Table 3.

The predictive performance of the LASSO-based Radscore, incorporating clinical indicators, was compared to that of the RF-based Radscore in conjunction with clinical indicators. These models were subsequently assessed against an SVM-based nomogram. In the training cohort, the LASSO-based Radscore achieved an AUC of 0.889 (95% CI: 0.824–0.954), while in the validation cohort, it yielded an AUC of 0.825 (95% CI: 0.722–0.928). Similarly, the RF-based Radscore, when utilized with clinical indicators, exhibited an AUC of 0.835 (95% CI: 0.748–0.922) in the training cohort and an AUC of 0.815 (95% CI: 0.709–0.922) in the validation cohort. For a more comprehensive assessment of the performance of these three models, please consult Figure S3.

The calibration curve of the radiomics nomogram displayed favorable agreement between the probabilities predicted by the nomogram and the actual probabilities of pCR observed in the training cohort. This favorable calibration and strong performance of the radiomics nomogram were confirmed in the validation cohort, as demonstrated in Figure 6C,D. The calibration plot for the nomogram showed closer alignment with the diagonal line compared to the other models, indicating a higher predictive accuracy.

Lastly, the decision curve analysis (DCA) revealed that the radiomics nomogram, incorporating both radiomics scores and clinical indicators, provided the highest clinical net benefit in both the training and validation cohorts. This suggests a strong performance of the nomogram in terms of its clinical applicability, as depicted in Figure 6E,F.

## 4. Discussion

In our study, we employed a radiomics approach to extract 851 quantitative imaging features from pretreatment contrast-enhanced planning CT scans. Subsequently, we developed and validated a radiomics nomogram that incorporates the planning CT-based Radscore and the selected clinical indicator (pretreatment T stage). The proposed nomogram could be used for the individualized prediction of the pCR before neoadjuvant CRT for the patients with LARC. This was consistent with the findings of Cui et al. [21] and Chen et al. [31].

To develop the Radscore, we employed the SVM methodology to select the 18 suitable radiomics predictors from the initial 851 candidate radiomics features. The Radscore demonstrated favorable prognostic performance for pCR in the training cohort, with AUC values of 0.880, which were subsequently validated in the validation cohort with AUC values of 0.830. Numerous studies showed a correlation between radiomics score and treatment response. Our study confirmed this association, as the radiomics scores derived from planning CT images were significantly higher in the pCR group, with all corresponding AUC values being greater than or nearly 80%. While some studies favored SVM classifiers over the LASSO or RF methods [32,33,34,35] in the development of radiomics models, other studies reported the superior performance of the LASSO or RF classifiers [27,36,37,38,39]. These divergent findings suggest that the choice of machine learning model influenced the predictive performance. In our study, the SVM method outperformed the LASSO or RF classifiers in the prediction of pCR to neoadjuvant chemoradiotherapy in LARC. This may be because the SVM model performed well in high-dimensional space [40,41]. Additionally, SVM classifiers could handle small datasets effectively [42], as they require only a small number of support vectors to define decision boundaries. Furthermore, SVM demonstrates robustness against noise [43], making it a reliable method for non-invasively characterizing intratumoral heterogeneity.

Limited literature exists on the use of CT-based radiomics to predict pCR to neoadjuvant CRT in LARC patients (refer to Table S5). Yuan et al. and Lutsyk et al. reported achieving an AUC of 0.872 and an accuracy of 0.839, respectively, for predicting pCR in LARC patients receiving neoadjuvant CRT using the RF method based on non-contrast-enhanced CT images [12,13]. Conversely, the model reported by Hamerla et al. [44] using the RF method showed no predictive power for treatment response. In our study, we utilized contrast-enhanced CT images for radiomic analysis, which should offer superior performance compared to non-contrast-enhanced images. Contrast enhancement enables the visualization of more intricate details regarding the heterogeneous internal architecture of malignant tumors. Previous results also support the use of radiomics based on contrast-enhanced CT images in predicting treatment response in LARC [14,15,16,17,18]. Bonomo et al. and Bibault et al. investigated the capability of radiomics features derived from contrast-enhanced CT for predicting pCR; their findings revealed AUC values of 0.63 and a predictive accuracy of 0.80 [14,15]. These values are significantly lower than the AUC values obtained in our study, which utilized a greater number of high-dimensional features, providing more detailed information on intratumoral heterogeneity. It is important to note that the absence of independent validation or small sample size in previous studies may have hindered the clinical applicability of their findings.

A recently established predictive model which incorporates clinical indicators and the LASSO-based Radscore has shown improved prognostic performance with an AUC of 0.822 [16]. Similarly, another predictive model utilizing the RF method, integrating clinical indicators, dose-volume histogram (DVH), and radiomics texture, achieved an AUC of 0.828 [17]. These findings align with Mao et al.’s study [18], where the integration of clinical indicators and Radscore via a nomogram successfully improved the prediction of pCR in patients with LARC using the LASSO method (AUC: 0.872). Consistent with these results, our study demonstrates that the integration of the SVM-based Radscore with clinical indicators (AUC: 0.866) yields superior performance when contrasted with both the LASSO method combined with clinical indicators (AUC: 0.825) and the RF method coupled with clinical indicators (AUC: 0.815) (see Figure S3). Our findings suggest that the SVM-based nomogram, characterized by a higher AUC and superior calibration, exhibits enhanced predictive efficacy compared to either the Radscore or clinical indicators in isolation. Consequently, our study introduces a radiomics nomogram as a readily applicable and valuable tool for the non-invasive analysis and characterization of rectal cancer.

However, the successful implementation of radiomics in clinical practice faces challenges, including the accurate segmentation and extraction of stable and comparable quantitative image features. Compared to MRI or PET/CT, planning CT images, readily available in radiation therapy departments, provide higher consistency and professionalism, as all tumor regions of interest (ROIs) were manually contoured by experienced radiation oncologists. Thus, the crucial factor lies in identifying a reliable feature selection method that maximizes the accuracy of the radiomics signature. To address this, our study applied various machine learning classifiers, including LASSO, RF and SVM, to the extracted features for key feature selection and generating a radiomics score in both the training and validation cohorts.

Despite these findings, our study exhibits several limitations. Firstly, the sample size of patients with pCR was small, with just 12 patients achieving a pCR in the validation cohort. This limitation is further compounded by the inherent challenge of an imbalanced dataset. Although we diligently addressed this imbalance through the application of diverse machine learning methods and conducted a comprehensive performance evaluation, it is evident that the model’s robustness may be affected. Secondly, our radiomics model only utilized CT images, warranting the further investigation of various modalities such as MRI, molecular biomarkers and gene expression. Moreover, our study solely utilized pre-CRT CT images rather than combining pre- and post-CRT images. Nevertheless, current studies in this field suffer from overall low quality and exhibit heterogeneity, limiting the robustness and replicability of the conclusions [45]. Lastly, as a retrospective study conducted in a single center, it is essential to validate our proposed model in a large-scale, independent, multicenter cohort to assess reproducibility and robustness.

## 5. Conclusions

A Radscore derived from SVM and based on pretreatment contrast-enhanced planning CT scans was developed for predicting pCR in patients with LARC. The Radscore exhibited promising performance in accurately predicting pCR. Moreover, the proposed nomogram, which incorporates the SVM-derived Radscore and clinical indicators, holds considerable value as a non-invasive tool for evaluating treatment outcomes in LARC patients. This approach has the potential to enhance clinical decision making and contribute to personalized patient management strategies.

## Figures and Tables

**Table 1:** Pretreatment characteristics of patients with pCR in the training and validation cohorts.

	Training Cohort			Validation Cohort		
Characteristics	Non-pCR	pCR	*p* Value	Non-pCR	pCR	*p* Value
Gender			0.819			0.960
Male	74 (67.3%)	27 (71.1%)		37 (72.5%)	8 (66.7%)	
Female	36 (22.7%)	11 (28.9%)		14 (27.5%)	4 (33.3%)	
Age			0.341			0.052
>60	49 (44.5%)	21 (55.3%)		27 (52.9%)	2 (16.7%)	
≤60	61 (55.5%)	17 (44.7%)		24 (47.1%)	10 (83.3%)	
BMI			0.056			0.687
>21.7	66 (60.0%)	30 (78.9%)		37 (72.5%)	10 (83.3%)	
≤21.7	44 (40.0%)	8 (21.1%)		14 (27.5%)	2 (16.7%)	
Tumor volume	110	38	0.005 *	51	12	0.914
>51.7 cm^3^	63 (57.3%)	11 (28.9%)		29 (56.9%)	6 (50.0%)	
≤51.7 cm^3^	47 (42.7%)	27 (71.1%)		22 (43.1%)	6 (50.0%)	
Tumor diameter			0.061			0.237
>4.7 cm	53 (48.2%)	11 (28.9%)		25 (49.0%)	3 (25.0%)	
≤4.7 cm	57 (51.8%)	27 (71.1%)		26 (51.0%)	9 (75.0%)	
Tumor length			0.093			0.934
>4.7 cm	79 (71.8%)	21 (55.3%)		35 (31.4%)	9 (75.0%)	
≤4.7 cm	31 (28.2%)	17 (44.7%)		16 (68.6%)	3 (25.0%)	
Distance from anal verge			0.356			0.898
>4.0 cm	86 (78.2%)	33 (86.8%)		39 (76.5%)	10 (83.3%)	
≤4.0 cm	24 (21.8%)	5 (13.2%)		12 (23.5%)	2 (16.7%)	
T stage			0.951			0.225
2	4 (3.4%)	1 (3.2%)		2 (3.9%)	0 (0.0%)	
3	59 (54.0%)	21 (61.9%)		29 (56.9%)	10 (83.3%)	
4	47 (42.6%)	16 (34.9%)		20 (39.2%)	2 (16.7%)	
N stage			0.650			0.627
0	9 (8.2%)	3 (7.9%)		8 (25.7%)	1 (8.3%)	
1	33 (30.0%)	12 (31.6%)		12 (23.5%)	5 (41.7%)	
2	37 (33.6%)	16 (42.1%)		26 (51.0%)	5 (41.7%)	
3	31 (28.2%)	7 (18.4%)		5 (9.8%)	1 (8.3%)	
CEA			1			0.237
>5 ng/mL	49 (44.5%)	17 (44.7%)		25 (49.0%)	3 (25.0%)	
≤5 ng/mL	61 (55.5%)	21 (55.3%)		26 (51.0%)	9 (75.0%)	
CA19-9			0.453			1
>37 U/mL	13 (11.8%)	7 (7.9%)		6 (11.8%)	1 (8.3%)	
≤37 U/mL	97 (88.2%)	31 (92.1%)		45 (88.2%)	11 (91.7%)	
Prescription dose			1			0.171
57.5 Gy	80 (72.7%)	27 (71.1%)		29 (56.9%)	10 (83.3%)	
50 Gy	30 (27.3%)	11 (28.9%)		22 (43.1%)	2 (16.7%)	
Radscore (mean ± SD)	−1.6742 ± 0.8722	−0.2601 ± 1.1362	0.000 *	−1.6379 ± 0.9098	−0.3133 ± 1.0479	0.000 *

pCR: pathological complete response; BMI: body mass index; CEA: carcinoembryonic antigen; CA19-9: carbohydrate antigen 19-9. * *p* < 0.05.

**Table 2:** Logistic regression analysis of risk factors for pCR.

	Univariate Analysis			Multivariate Analysis		
Features	β	OR (95% CI)	*p* Value	β	OR (95% CI)	*p* Value
Gender	0.177	1.194 (0.543–2.757)	0.666			
Age	0.430	1.538 (0.734–3.261)	0.255			
BMI	0.916	2.500 (1.090–6.307)	0.039 *			
Tumor volume	−1.191	0.304 (0.133–0.659)	0.003 *			
Tumor diameter	−0.825	0.438 (0.191–0.949)	0.042 *			
Tumor length	−0.724	0.485 (0.226–1.045)	0.063			
Distance from anal verge	0.611	1.842 (0.694–5.825)	0.251			
T stage	−1.013	0.363 (0.137–0.595)	0.035 *	−1.167	0.311 (0.104–0.841)	0.027 *
N stage	−0.128	0.880 (0.588–1.317)	0.533			
CEA	0.008	1.008 (0.476–2.114)	0.984			
CA19-9	0.522	1.685 (0.588–4.505)	0.308			
Prescription dose	−0.011	0.989 (0.889–1.107)	0.842			
Radscore	1.451	4.266 (2.658–7.470)	0.000 *	1.651	5.212 (2.993–10.161)	0.000 *

pCR: pathological complete response; β: regression coefficient; OR: odds ratio; CI: confidence interval; BMI: body mass index; CEA: carcinoembryonic antigen; CA19-9: carbohydrate antigen 19-9. * *p* < 0.05.

**Table 3:** Performance evaluation of three predictive models.

	Clinical		Radiomics		Nomogram	
Metrics	Training (95% CI)	Validation (95% CI)	Training (95% CI)	Validation (95% CI)	Training (95%CI)	Validation (95% CI)
AUC	0.770 (0.629–0.911)	0.725 (0.642–0.808)	0.880 (0.823–0.946)	0.830 (0.722–0.928)	0.910 (0.815–0.976)	0.866 (0.762–0.970)
Accuracy	0.750 (0.676–0.764)	0.683 (0.540–0.857)	0.851 (0.703–0.912)	0.810 (0.667–0.873)	0.885 (0.797–0.946)	0.841 (0.651–0.937)
Sensitivity	0.605 (0.553–0.842)	0.750 (0.500–1)	0.711 (0.632–1)	0.917 (0.750–1)	0.816 (0.684–0.947)	0.917 (0.667–1)
Specificity	0.800 (0.554–0.918)	0.667 (0.471–0.902)	0.900 (0.627–0.964)	0.784 (0.588–0.882)	0.909 (0.763–0.991)	0.824 (0.588–0.961)
PPV	0.511 (0.343–0.786)	0.346 (0.250–0.611)	0.711 (0.462–0.879)	0.500 (0.344–0.611)	0.756 (0.581–0.968)	0.550 (0.333–0.833)
NPV	0.854 (0.815–0.926)	0.919 (0.857–0.975)	0.900 (0.880–0.989)	0.976 (0.921–1)	0.935 (0.897–0.980)	0.977 (0.907–1)
MCC	0.385 (0.094–0.742)	0.332 (0.023–0.798)	0.611 (0.228–0.934)	0.577 (0.266–0.766)	0.708 (0.409–0.947)	0.624 (0.201–0.908)
F1 score	0.554 (0.423–0.813)	0.474 (0.333–0.759)	0.711 (0.534–0.936)	0.647 (0.472–0.759)	0.785 (0.628–0.957)	0.688 (0.444–0.909)

AUC: area under the receiver operator characteristic curves; NPV: negative-predictive value, PPV: positive-predictive value; CI: confidence interval; MCC: Matthew’s correlation coefficient.

## Data Availability

The datasets utilized in the present study can be obtained from the corresponding author upon reasonable request.

## Supplementary Materials

The following supporting information can be downloaded at: https://www.mdpi.com/article/10.3390/cancers15215134/s1. Supplementary materials are available regarding the patient recruitment pathway, radiomic features, and Radscore construction, providing further details beyond the initial descriptions. Figure S1: Patient recruitment pathway of LARC who received neoadjuvant CRT; Figure S2: Radiomic feature coefficients of three radiomics classifiers; Figure S3: Predictive performance evaluation of three methods: LASSO, RF and SVM, when combined with clinical indicators; Table S1: Pretreatment clinical characteristics of patients with LARC; Table S2: Performance evaluation of three radiomics classifiers; Table S3: Radiomics score calculation formulas of three radiomics classifiers; Table S4: Summary of radiomic features; Table S5: Summary of differences between our study and previous radiomics studies predicting pCR status in rectal cancer based on CT imaging.
