# Supplementary material for: Radiomics Signature Based on Support Vector Machines for the Prediction of Pathological Complete Response to Neoadjuvant Chemoradiotherapy in Locally Advanced Rectal Cancer
Source: Cancers (Basel). 2023 Oct 25;15(21):5134. doi: 10.3390/cancers15215134 (PMC10648149; doi:10.3390/cancers15215134)
Supplement: Supplementary file 1 [file cancers-15-05134-s001.zip › cancers-2644398-supplementary.pdf]

Supplementary Materials

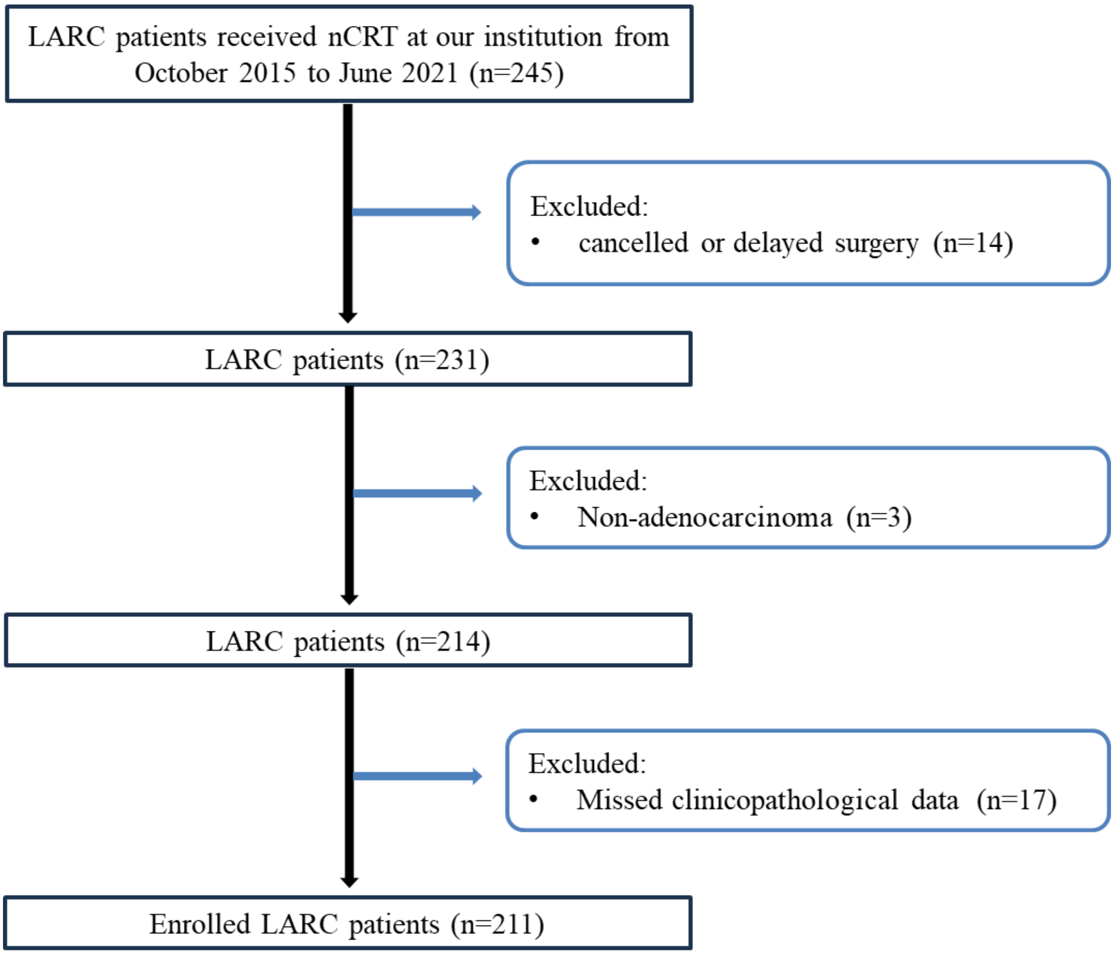

Figure S1 Patient recruitment pathway of LARC who received neoadjuvant CRT. LARC: locally advanced rectal cancer; nCRT: neoadjuvant chemoradiotherapy.

A

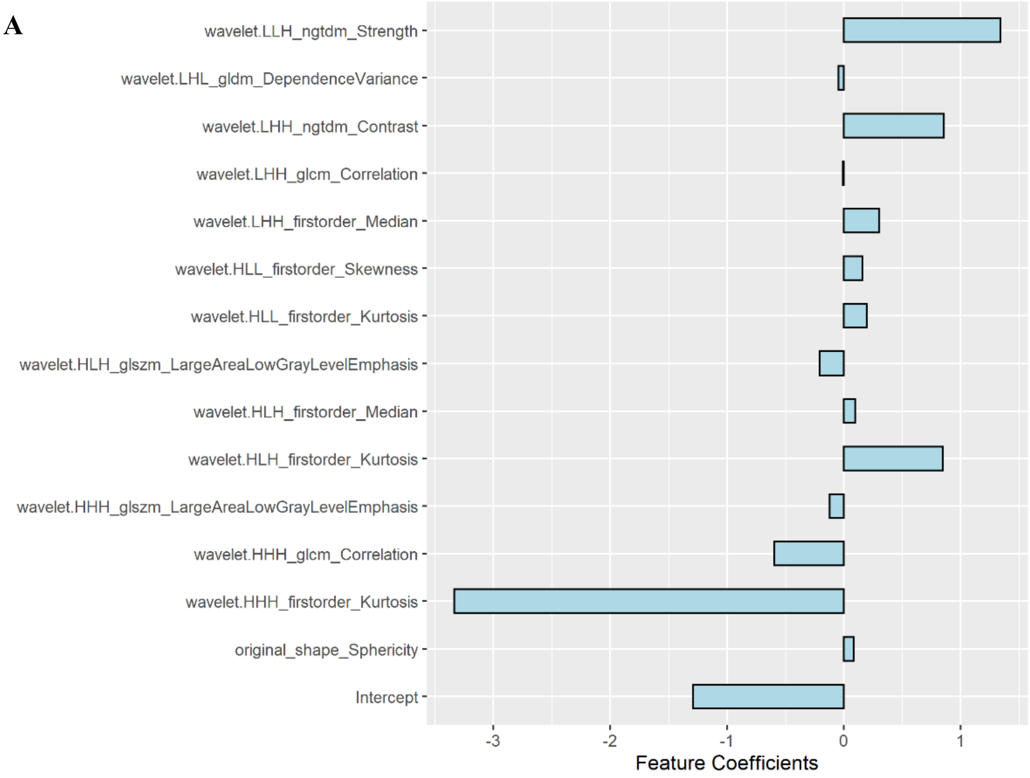

wavelet.LLH_ngtdm_Strength
wavelet.LHL_gldm_DependenceVariance
wavelet.LHH_ngtdm_Contrast
wavelet.LHH_glcm_Correlation
wavelet.LHH_firstorder_Median
wavelet.HLL_firstorder_Skewness
wavelet.HLL_firstorder_Kurtosis
wavelet.HLH_glszm_LargeAreaLowGrayLevelEmphasis
wavelet.HLH_firstorder_Median
wavelet.HLH_firstorder_Kurtosis
wavelet.HHH_glszm_LargeAreaLowGrayLevelEmphasis
wavelet.HHH_glcm_Correlation
wavelet.HHH_firstorder_Kurtosis
original_shape_Sphericity
Intercept
-3
-2
-1
0
1
Feature Coefficients

B

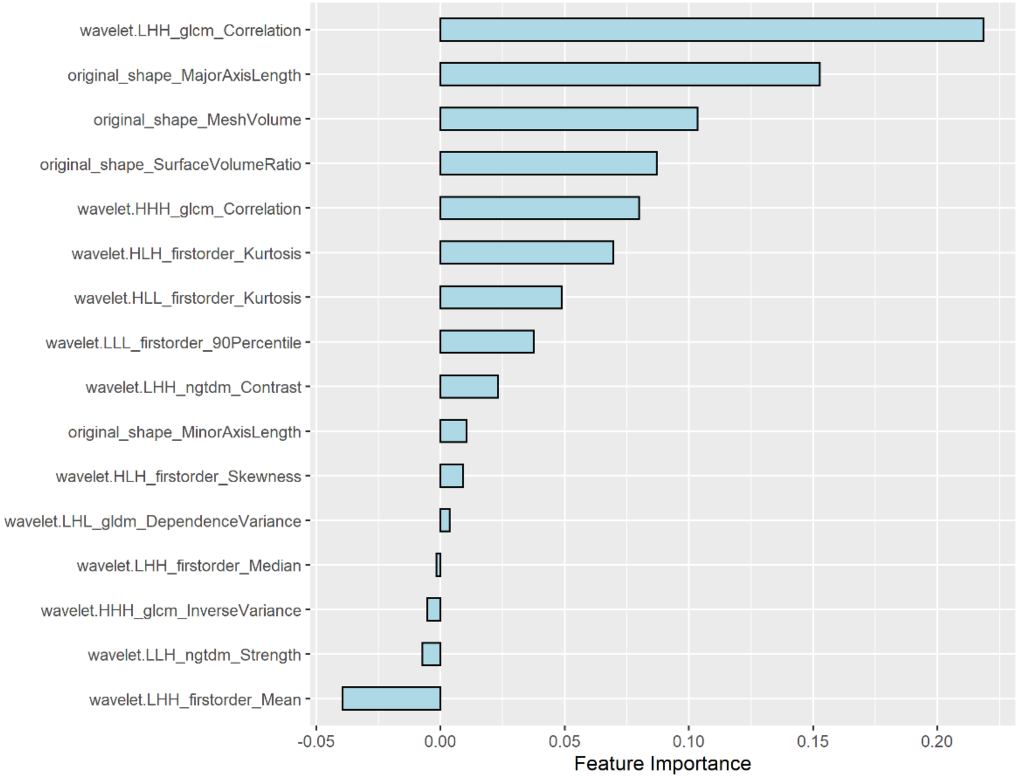

wavelet.LHH_glcm_Correlation
original_shape_MajorAxisLength
original_shape_MeshVolume
original_shape_SurfaceVolumeRatio
wavelet.HHH_glcm_Correlation
wavelet.HLH_firstorder_Kurtosis
wavelet.HLL_firstorder_Kurtosis
wavelet.LLL_firstorder_90Percentile
wavelet.LHH_ngtdm_Contrast
original_shape_MinorAxisLength
wavelet.HLH_firstorder_Skewness
wavelet.LHL_gldm_DependenceVariance
wavelet.LHH_firstorder_Median
wavelet.HHH_glcm_InverseVariance
wavelet.LLH_ngtdm_Strength
wavelet.LHH_firstorder_Mean
-0.05
0.00
0.05
0.10
0.15
0.20
Feature Importance

C

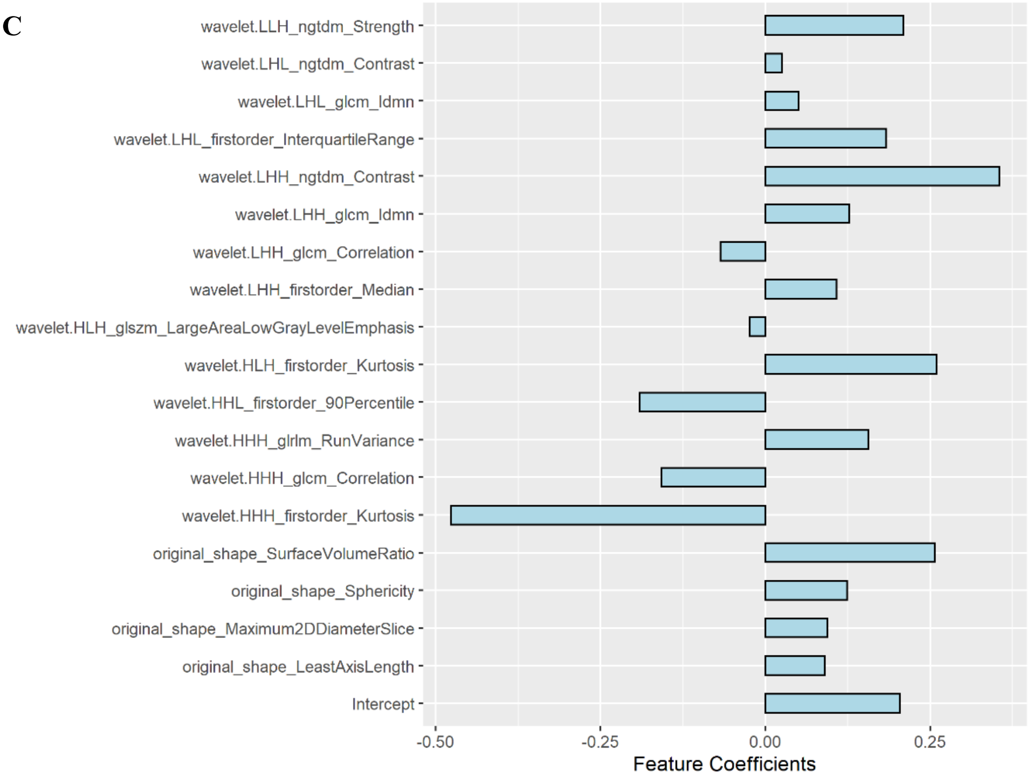

Figure S2 Radiomics feature coefficients of three radiomics classifiers. (A) Radiomics feature coefficients were determined based on LASSO, enabling the calculation of the Radscore. (B) Radiomics feature coefficients were obtained based on RF, allowing for the calculation of the Radscore. (C) Radiomics feature coefficients were obtained using SVM, enabling the calculation of the Radscore. LASSO: least absolute shrinkage and selection operator; RF: random forest; SVM: support vector machines.

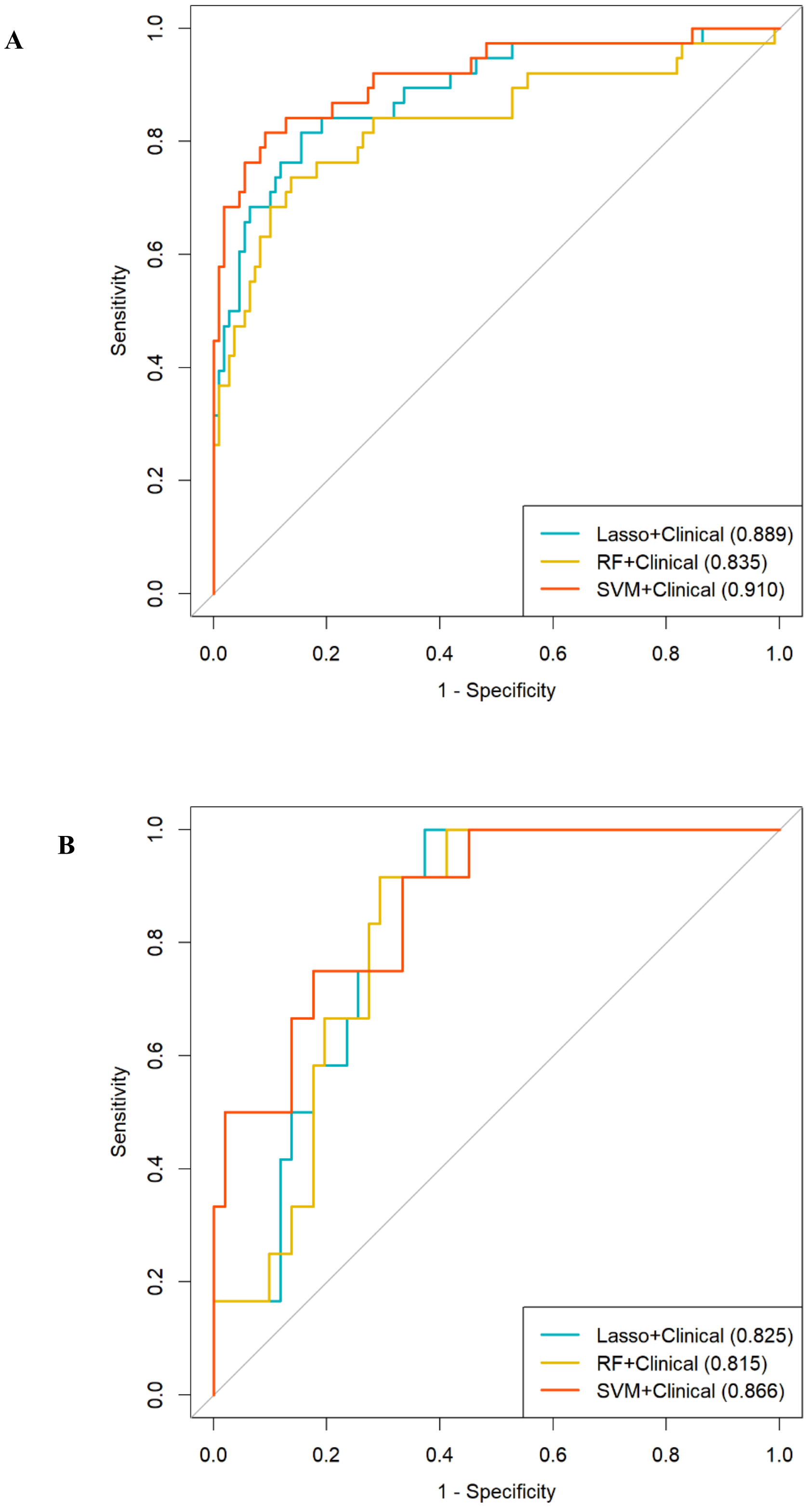

Figure S3. Predictive performance evaluation of three methods: LASSO, RF and SVM, when combined with clinical indicators. (A) ROC curves were presented to visualize the performance within the training cohort. (B) ROC curves were provided to assess performance in the validation cohort. LASSO: least absolute shrinkage and selection operator; RF: random forest; SVM: support vector machines; ROC: receiver operating characteristic.

Table S1 Pretreatment clinical characteristics of patients with LARC

**Characteristics**	**Training**	**Validation**	**P value**
**Gender**			0.767
**Male**	101 (68.2%)	45 (71.4%)	
**Female**	47 (31.8.0%)	18 (28.6%)	
**Age**			0.986
**> 60**	70 (47.3%)	29 (46.0%)	
**≤ 60**	78 (52.7%)	34 (54.0%)	
**BMI**			0.221
**> 21.7**	96 (64.9%)	47 (74.6%)	
**≤ 21.7**	52 (35.1%)	16 (25.4%)	
**Tumor volume**			0.556
**> 51.7 cm^3**	74 (50.0%)	35 (55.6%)	
**≤ 51.7 cm^3**	74 (50.0%)	28 (44.4%)	
**Tumor diameter**			0.993
**> 4.7 cm**	64 (43.2%)	28 (44.4%)	
**≤ 4.7 cm**	84 (56.8%)	35 (55.6%)	
**Tumor length**			0.870
**> 4.7 cm**	100 (67.6%)	44 (69.8%)	
**≤ 4.7 cm**	48 (32.4%)	19 (30.2%)	
**Distance from anal verge**			0.805
**> 4 cm**	119 (80.4%)	49 (77.8%)	
**≤ 4 cm**	29 (19.6%)	14 (22.2%)	
**T stage**			0.568
**2**	5 (3.4%)	2 (3.2%)	
**3**	80 (54.0%)	39 (61.9%)	
**4**	63 (42.6%)	22 (34.9%)	
**N stage**			0.025
**0**	12 (8.1%)	9 (14.3%)	
**1**	45 (30.4%)	17 (27.0%)	
**2**	53 (35.8%)	31 (49.2%)	
**3**	38 (25.7%)	6 (9.5%)	
**CEA**			1
**> 5 ng/mL**	66 (44.6%)	28 (44.4%)	
**≤ 5 ng/mL**	82 (55.4%)	35 (55.6%)	
**CA19-9**			0.800
**> 37 U/mL**	20 (13.5%)	7 (11.1%)	
**≤ 37 U/mL**	128 (86.5%)	56 (88.9%)	
**Prescription dose**			0.182
**57.5 Gy**	107 (72.3%)	39 (61.9%)	
**50 Gy**	41 (27.7%)	24 (38.1%)	
**Radscore (mean ± SD)**	-1.3111 ± 1.1283	-1.2505 ± 2.4737	0.853

pCR: pathological complete response; BMI: body mass index; CEA: carcinoembryonic antigen; CA19-9: carbohydrate antigen 199.

Table S2 Performance evaluation of three radiomics classifiers

	LASSO		RF		SVM	
**Metrics**	**Training (95%CI)**	**Validation (95%CI)**	**Training (95%CI)**	**Validation (95%CI)**	**Training (95%CI)**	**Validation (95%CI)**
**AUC**	0.841 (0.758-0.924)	0.806 (0.696-0.916)	0.818 (0.734-0.902)	0.791 (0.670-0.912)	0.880 (0.823-0.946)	0.830 (0.722-0.928)
**Accuracy**	0.851 (0.777-0.926)	0.762 (0.635-0.873)	0.824 (0.628-0.892)	0.730 (0.587-0.857)	0.851 (0.703-0.912)	0.810 (0.667-0.873)
**Sensitivity**	0.658 (0.553-0.868)	0.917 (0.750-1)	0.605 (0.474-0.947)	0.917 (0.667-1)	0.711 (0.632-1)	0.917 (0.750-1)
**Specificity**	0.918 (0.764-0.991)	0.725 (0.588-0.863)	0.900 (0.546-0.982)	0.686 (0.529-0.863)	0.900 (0.627-0.964)	0.784 (0.588-0.882)
**PPV**	0.735 (0.537-0.958)	0.440 (0.333-0.600)	0.676 (0.412-0.903)	0.407 (0.294-0.588)	0.711 (0.462-0.879)	0.500 (0.344-0.611)
**NPV**	0.886 (0.856-0.949)	0.974 (0.911-1)	0.868 (0.852-0.941)	0.972 (0.892-1)	0.900 (0.880-0.989)	0.976 (0.921-1)
**MCC**	0.598 (0.298-0.892)	0.515 (0.266-0.739)	0.525 (0.018-0.929)	0.478 (0.154-0.739)	0.611 (0.228-0.934)	0.577 (0.266-0.766)
**F1 score**	0.694 (0.545-0.911)	0.595 (0.461-0.750)	0.639 (0.441-0.924)	0.564 (0.408-0.741)	0.711 (0.534-0.936)	0.647 (0.472-0.759)

LASSO: least absolute shrinkage and selection operator; RF: random forest; SVM: support vector machines; AUC: area under the receiver operating characteristic curve; NPV: negative-predictive value, PPV: positive-predictive value; CI: confidence interval; MCC: matthews correlation coefficient.

Table S3 Radiomics score calculation formulas of three radiomics classifiers

**LASSO Radscore** = −1.2930 + 0.0856*original_shape_Sphericity + 1.3395*wavelet.LLH_ngtdm_Strength − 0.0464*wavelet.LHL_gldm_DependenceVariance + 0.3033*wavelet.LHH_firstorder_Median − 0.0096*wavelet.LHH_glcm_Correlation + 0.8562*wavelet.LHH_ngtdm_Contrast + 0.1960*wavelet.HLL_firstorder_Kurtosis + 0.1592*wavelet.HLL_firstorder_Skewness + 0.8433*wavelet.HLH_firstorder_Kurtosis + 0.0954*wavelet.HLH_firstorder_Median − 0.2079*wavelet.HLH_glszm_LargeAreaLowGrayLevelEmphasis − 3.3334*wavelet.HHH_firstorder_Kurtosis − 0.5956*wavelet.HHH_glcm_Correlation − 0.1212*wavelet.HHH_glszm_LargeAreaLowGrayLevelEmphasis
**RF Radscore** = 0.2185*wavelet.LHH_glcm_Correlation + 0.1527*original_shape_MajorAxisLength + 0.1035*original_shape_MeshVolume + 0.0799*wavelet.HHH_glcm_Correlation + 0.0871*original_shape_SurfaceVolumeRatio + 0.0696*wavelet.HLH_firstorder_Kurtosis + 0.0037*wavelet.LHL_gldm_DependenceVariance + 0.0232*wavelet.LHH_ngtdm_Contrast + 0.0090*wavelet.HLH_firstorder_Skewness − 0.0395*wavelet.LHH_firstorder_Mean + 0.0104*original_shape_MinorAxisLength − 0.0054*wavelet.HHH_glcm_InverseVariance − 0.0074*wavelet.LLH_ngtdm_Strength + 0.0374*wavelet.LLL_firstorder_90Percentile – 0.0017*wavelet.LHH_firstorder_Median + 0.0488*wavelet.HLL_firstorder_Kurtosis
**SVM Radscore** = 0.2039 + 0.3550*wavelet.LHH_ngtdm_Contrast − 0.1573*wavelet.HHH_glcm_Correlation + 0.2601*wavelet.HLH_firstorder_Kurtosis + 0.1559*wavelet.HHH_glrlm_RunVariance + 0.2574*original_shape_SurfaceVolumeRatio + 0.0943*original_shape_Maximum2DDiameterSlice + 0.2096*wavelet.LLH_ngtdm_Strength + 0.1083*wavelet.LHH_firstorder_Median + 0.1243*original_shape_Sphericity − 0.47707215*wavelet.HHH_firstorder_Kurtosis − 0.1908*wavelet.HHL_firstorder_90Percentile + 0.0256*wavelet.LHL_ngtdm_Contrast + 0.1833*wavelet.LHL_firstorder_InterquartileRange + 0.1277* wavelet.LHH_glcm_Idmn − 0.0679*wavelet.LHH_glcm_Correlation + 0.0897*original_shape_LeastAxisLength + 0.0508*wavelet.LHL_glcm_Idmn − 0.0241*wavelet.HLH_glszm_LargeAreaLowGrayLevelEmphasis

LASSO: least absolute shrinkage and selection operator; RF: random forest; SVM: support vector machines

Table S4 Summary of radiomic features

Feature class	Feature name	Number
**Shape**	Elongation, Least Axis Length, Major Axis Length, Maximum2DDiameterColumn, Maximum2DDiameterRow, Maximum2DDiameterSlice, Maximum 3D Diameter, Mesh Volume, Minor Axis Length, Sphericity, Surface Area, Surface Volume Ratio, Flatness, Voxel Volum.	14
**First-order**	10Percentile, 90Percentile, Energy, Entropy, Interquartile Range, Kurtosis, Maximum,Mean Absolute Deviation, Mean, Median, Minimum, Range, Robust Mean Absolute Deviation, Uniformity, Root Mean Squared, Skewness, Total Energy, Variance.	18
**GLCM**	Autocorrelation, Cluster Prominence, Cluster Shade, Cluster Tendency, Contrast, Correlation,Difference Average, Difference Entropy, Difference Variance, Id, Idm, Idmn, Idn, Imc1, Imc2, Inverse Variance, Joint Average, Joint Energy, Joint Entropy, MCC, Maximum Probability, Sum Average, Sum Entropy, Sum Squares	24
**GLDM**	Dependence Entropy, Dependence NonUniformity, Dependence NonUniformity Normalized, Dependence Variance, Gray Level NonUniformity, Gray Level Variance, High Gray Level Emphasis, Large Dependence Emphasis, Large Dependence High Gray Level Emphasis, Large Dependence Low Gray Level Emphasis, Low Gray Level Emphasis, Small Dependence Emphasis, Small Dependence High Gray Level Emphasis, Small Dependence Low Gray Level Emphasis.	14
**GLRLM**	Gray Level Non Uniformity, Gray Level Non Uniformity Normalized, Long Run Emphasis, High Gray Level Run Emphasis, Long Run High Gray Level Emphasis, Gray Level Variance, Run Entropy, Long Run Low Gray Level Emphasis, Low Gray Level Run Emphasis, Run Length Non Uniformity, Run Length Non Uniformity Normalized, Run Percentage, Run Variance, Short Run Emphasis, Short Run High Gray Level Emphasis, Short Run Low Gray Level Emphasis.	16
**GLSZM**	Gray Level Non Uniformity, Gray Level Non Uniformity Normalized, Gray Level Variance, High Gray Level Zone, Emphasis, Large Area Emphasis, Large Area High Gray Level Emphasis, Large Area Low Gray Level Emphasis, Low Gray Level Zone Emphasis, Size Zone Non Uniformity, Size Zone Non Uniformity Normalized, Small Area Emphasis, Small Area High Gray Level Emphasis, Small Area High Gray Level Emphasis, Zone Entropy, Zone Percentage, Zone Variance.	16
**NGTDM**	Busyness, Coarseness, Complexity, Contrast, Strength	5
**Wavelet**	Wavelet filtering employs a sophisticated multi-scale decomposition technique utilizing wavelet functions, resulting in eight unique decompositions at each level. These decompositions are obtained by applying various combinations of High pass and Low pass filters in three dimensions: x, y, and z. The resulting combinations are denoted as HHH, HHL, HLH, HLL, LHH, LHL, LLH and LLL.	744

GLCM: grey-level co-occurrence matrix; GLRLM: grey-level run length matrix; GLSZM: gray-level size zone matrix; GLDM: gray-level dependence matrix features; NGTDM: neighbor gray-level difference matrix

Table S5 Summary of differences between our study and previous radiomics studies predicting pCR status in rectal cancer based on CT imaging

**Literature**	**Modality**	**Patient numbers**	**Features**	**Feature selection (radiomics signature)**	**AUC**
Yuan et al[12]	Non-contrast enhanced CT	91	Radiomics	RF	0.839 (Accuracy)
Lutsyk et al[13]	Non-contrast enhanced CT	140	Radiomics wavelets	RF (no Radscore construction)	0.872
Bonomo et al[14]	Contrast-enhanced CT	201	Radiomics	KNN	0.63
Bibault et al[15]	Contrast CT	95	Radiomics	DNN (no Radscore construction)	0.80 (Accuracy)
Zhuang et al[16]	Contrast-enhanced CT	177	Radiomics + Clinical	LASSO	0.822
Wang et al[17]	CT	217	Radiomics Texture + DVH + Clinical	RF (no Radscore construction)	0.828
Mao et al[18]	Contrast-enhanced CT	216	Radiomics + Clinical	LASSO	0.872
**Our study**	Contrast-enhanced CT	211	Radiomics + Clinical	LASSO, RF, SVM	0.866

LASSO: least absolute shrinkage and selection operator; RF: random forest; SVM: support vector machines; KNN: k-nearest neighbor; DNN: deep neural network
